# Cerebrospinal fluid sTREM2 in Alzheimer’s disease: comparisons between clinical presentation and AT classification

**DOI:** 10.1038/s41598-020-72878-8

**Published:** 2020-09-28

**Authors:** Anne-Brita Knapskog, Kristi Henjum, Ane-Victoria Idland, Rannveig Sakshaug Eldholm, Karin Persson, Ingvild Saltvedt, Leiv Otto Watne, Knut Engedal, Lars N. G. Nilsson

**Affiliations:** 1 Department of Geriatric Medicine, Memory Clinic, Oslo University Hospital, Ullevaal, Postboks 4956, Nydalen, 0424 Oslo, Norway; 2 Department of Pharmacology, Institute of Clinical Medicine, University of Oslo and Oslo University Hospital, Oslo, Norway; 3 Department of Geriatric Medicine, Institute of Clinical Medicine, University of Oslo, Oslo, Norway; 4 Institute of Basic Medical Sciences, University of Oslo, Oslo, Norway; 5 Department of Neuromedicine and Movement Science, Norwegian University of Science and Technology (NTNU), Trondheim, Norway; 6 Department of Geriatrics, St Olavs Hospital, University Hospital of Trondheim, Trondheim, Norway; 7 Norwegian National Advisory Unit On Ageing and Health, Vestfold Hospital Trust, Tønsberg, Norway

**Keywords:** Biomarkers, Diseases, Medical research, Neurology

## Abstract

Triggering receptor expressed on myeloid cells 2 (TREM2) is an innate immune receptor expressed by microglia. Its cleaved fragments, soluble TREM2 (sTREM2), can be measured in the cerebrospinal fluid (CSF). Previous studies indicate higher CSF sTREM2 in symptomatic AD; however most of these studies have included biomarker positive AD cases and biomarker negative controls. The aim of the study was to explore potential differences in the CSF level of sTREM2 and factors associated with an increased sTREM2 level in patients diagnosed with mild cognitive impairment (MCI) or dementia due to AD compared with cognitively unimpaired controls as judged by clinical symptoms and biomarker category (AT). We included 299 memory clinic patients, 62 (20.7%) with AD-MCI and 237 (79.3%) with AD dementia, and 113 cognitively unimpaired controls. CSF measures of the core biomarkers were applied to determine AT status. CSF sTREM2 was analyzed by ELISA. Patients presented with comparable CSF sTREM2 levels as the cognitively unimpaired (9.6 ng/ml [SD 4.7] versus 8.8 ng/ml [SD 3.6], *p* = 0.27). We found that CSF sTREM2 associated with age-related neuroinflammation and tauopathy irrespectively of amyloid β, APOE ε4 status or gender. The findings were similar in both symptomatic and non-symptomatic individuals.

## Introduction

Alzheimer’s disease (AD) is the most common cause of dementia worldwide, and the neuropathological hallmarks are proteinaceous deposits of extracellular amyloid β (Aβ) deposits and intraneuronal neurofibrillary tangles (NFTs) and brain atrophy^1^. Establishment of biomarkers reflecting these changes have enabled an antemortem determination of the neuropathology, but much remains to be elucidated. In 2011, National Institute on Aging and Alzheimer’s Association (NIA–AA) revised the diagnostic criteria by incorporating AD diagnostic biomarkers to enhance specificity e.g. for research studies or clinical trials^2^. However, the clinical criteria remained the cornerstone of the AD diagnosis according to the NIA/AA^2^.

Currently a subclassification according to biomarker positivity is commonly applied by researchers and specialists^3^. The commonly found neuropathological changes are categorized into three groups—biomarkers reflecting amyloid-β pathology (A)_,_ phosphorylated tau (P-tau) pathology (T), and neurodegeneration (N)—referred to as the AT(N) classification. From classifying AD into clinical stages, there has been a shift—not only in research studies but also in clinical practice—to think of AD as a continuum of pathological changes beginning in cognitively unimpaired and ending with severe dementia^3^. However, as stated by Jack et al. it is too early and not appropriate to apply this framework in clinical practice^3^.

In recent years, it has become clear that neuroinflammation plays an important role in the pathogenesis of AD^4^. The microglia mediate immune responses in the brain and express the triggering receptor expressed on myeloid cells 2 (TREM2). In 2013, *TREM2* was found genetically linked to AD^5–9^ and additional rare variants of *TREM2* have since been associated with AD, strongly implicating a pathogenic role of TREM2-biology^10^. TREM2 relates to important microglial functions, including cytokine release, phagocytosis, proliferation and migration^11^. The AD-associated *TREM2* variants seem to cause loss of function of TREM2, postulated to reduce the ability of microglia to respond to toxic metabolites and clear them from the brain^12^. Genetic linkage of TREM2 variants to other dementias reinforces the importance of TREM2-function for cognitively normal aging^13,14^.

The transmembrane TREM2 receptor undergoes ectodomain shedding, leading to release of soluble fragments, soluble TREM2 (sTREM2). In the cerebrospinal fluid (CSF) sTREM2 serves as a surrogate measure of microglial activity^15–17^. The value of CSF sTREM2 as an AD diagnostic biomarker has been explored in several studies^18–24^. Most studies show that CSF sTREM2 is increased in the AD continuum, but the results are somewhat inconsistent regarding disease stages^10^. Some studies indicate sTREM2 to increase in the preclinical phase while others find highest sTREM2 levels in the MCI stage of AD, indicating TREM2-related microglia activation in the early clinical stages of the disease^19,20,23,25^. Others report increased levels of sTREM2 in AD dementia^20–22^, decreased levels^16^ of sTREM2 in AD dementia, or no differences along the AD continuum^23,26^.

Mutations in the *TREM2* gene confer increased risk for AD, and the microglia harbor different cell-surface receptors that respond to Aβ-aggregates activating inflammatory processes^27–29^. There are however inconstancies in the association between CSF sTREM2 and Aβ_1-42_ (Aβ_42_). Some studies find an association between sTREM2 and Aβ_42_, but only in the cognitively healthy older control groups^19,20,23^, while one study also observed an association in younger patients with dementia^30^. Furthermore, no association has been found between sTREM2 and *APOE ε4* genotype^20,26^. In contrast, a positive association between sTREM2 and the biomarkers of tau pathology and neurodegeneration is more consistently reported^21–23,25,31–33^, supporting the hypothesis of sTREM2 being a microglial marker related to neurodegeneration and/or tauopathy.

In most published sTREM2 studies, AD patients and reference groups have been stratified according to their biomarker profile. There is also a need for complementary studies of AD cohorts with different clinical presentations to evaluate the usefulness of sTREM2 as a biomarker for diagnosing AD as a clinical syndrome in daily practice.

## Aim

By including a relatively large number of patients referred to two memory clinics for a diagnostic workup and cognitively unimpaired controls, we aimed to explore potential differences in sTREM2 levels in patients clinically diagnosed with AD at MCI or dementia stage relative to cognitively unimpaired, and the differences across and within these groups when defined by AT(N) category. We also aimed to further explore factors associated with the levels of sTREM2 in the study population.

## Methods

All methods in this study were carried out in accordance with relevant guidelines and regulations.

### Study design

This was a cross-sectional study with patients from two memory clinics in Norway (176 patients from Oslo University Hospital [OUH] and 143 patients from St. Olav University Hospital, Trondheim) included in the Norwegian Registry of Persons Assessed for Cognitive Symptoms (NorCog), and 113 cognitively unimpaired controls recruited from surgical departments at OUH and Diakonhjemmet Hospital, Oslo.

### Memory clinic patients

The patients were included in the study between June 2009 and September 2016 at their first regular visit to one of the memory clinics. Patients diagnosed with AD at MCI or dementia stage that went through lumbar puncture were included. Patients with dementia disorders due to other causes were excluded (including among others Lewy body dementia, frontotemporal dementia and Parkinson’s disease dementia).

### Assessments

All patients were assessed according to a comprehensive standardized research protocol by experienced physicians^34^. Information was obtained from the patients, caregivers, the patients’ general practitioners, and from medical records, and included demographic information on age, gender, years of education, and living situation. Previous disorders and medications in use were registered. Several cognitive tests were performed, including the Mini-Mental State Examination (MMSE), the Consortium to Establish a Registry of Alzheimer’s Disease (CERAD), 10-item word list and figure copying, the Clock Drawing Test (CDT), and the Trail Making Tests A and B (TMT A and B). The patients also went through a physical examination, including computed tomography (CT) or magnetic resonance imaging (MRI) brain scans, blood sampling, and lumbar puncture. AD CSF core biomarkers were analyzed at the laboratory at Akershus University Hospital (AHUS) using the INNOTEST enzyme-linked immunosorbent assays (ELISA; Fujirebio, Ghent, Belgium) for all three biomarkers. APOE genotyping using the Illumina Infinium OmniExpress v1.1 chip was carried out at deCODE Genetics (Reykjavik, Iceland).

### Diagnostic workup

All available data at baseline were used when the study researchers diagnosed the patients. However, information about *APOE* genotype status was obtained later and therefore not used in the diagnostic workup. The clinical diagnosis of MCI or dementia due to AD was based on the National Institutes of Health and the Alzheimer’s Association (NIA/AA) criteria including both patients with probable and possible AD^2,35^. Patients with an etiologically AD mixed cerebrovascular disease were included as well, but patients with all other mixed presentations were excluded. Patients with depression or other comorbidities known to influence the cognitive performance were also excluded. The NogCog registry arranges consensus conferences yearly as to harmonize diagnostic classification of patients at the different hospitals in Norway. In addition, the two memory clinics in the present study arrange weekly diagnostic consensus meetings to harmonize the diagnoses where the researchers participate as well.

### Cognitively unimpaired controls

In addition, a group of 113 cognitively unimpaired controls 65 years or older was included from surgical departments at OUH and Diakonhjemmet Hospital, Oslo, where they had been referred for elective surgery in spinal anesthesia due to gynecological, orthopedic, or urological problems. These went through the same cognitive test battery as the patients and only individuals with normal test results (age and education adjusted) at baseline were included. The majority were tested again after two years, and those who showed a decrease of more than 1 standard deviation (SD) in test results were excluded (applied for two participants). For further information about the cognitively unimpaired, see the previous publication^36^. CSF was collected from the cognitively unimpaired controls during the spinal anesthesia procedure, before the administration of the anesthetic and analyzed at Sahlgrenska University Hospital (Mölndal, Sweden) using INNOTEST enzyme-linked immunosorbent assays (ELISA; Fujirebio, Ghent, Belgium).

### AT(N) classification

According to the AT(N) research framework, the neuropathological changes commonly found are categorized into three groups: biomarkers reflecting amyloid-β pathology (A) including amyloid PET-imaging or low CSF Aβ42, phosphorylated tau (P-tau) pathology (T) including CSF P_181_-tau (P-tau) or tau-PET, and neurodegeneration (N) including CSF total tau (T-tau), FDG-PET or MRI-measured brain atrophy^3^. In the present study the cohort was categorized into profiles according to the AT(N) classification applying the CSF AD core biomarkers (Supplementary Tables 1 and 2). Low CSF Aβ_42_ was classified as “A+”, and high CSF P-tau as “T+”. As T-tau reflecting neuronal injury (“N”) was highly correlated with the P-tau, T-tau (N) was excluded from the regression analyses, resulting in four different profiles (A−T−, A+T−, A+T+ and A−T+). Although MRI examinations were performed on the majority of the patients, these had been executed and evaluated on different locations using different protocols and, therefore, it was not possible to include those results in this study.

The CSF levels of AD core markers for the memory clinic patients and the cognitive unimpaired controls were analyzed by the same kits but at two different laboratories (see memory clinic patients and cognitively unimpaired controls). Due to the known variability between different laboratories, each laboratory’s own cut-offs for pathological levels had to be applied as appropriate^37^. The following cut-off values were provided by the Norwegian laboratory and applied for the memory clinic patients: Aβ_42_ > 700 pg/ml and P-tau < 80 pg/ml. Age-adjusted cut-off levels for T-tau < 300 pg/ml for patients under the age of 50, < 450 pg/ml for those aged 50–70 years, and < 500 pg/ml for those older than 70 years. The cut-off values for the Swedish laboratory analyzing the cognitively unimpaired were Aβ_42_ > 530 pg/ml, P-tau < 60 pg/ml, and T-tau < 350 pg/ml ^38^.

### CSF sTREM2 measurements

The CSF from both patient and cognitively unimpaired cohorts were collected, centrifuged and the supernatant divided in aliquots and stored in polypropylene tubes at − 80 °C. All CSF sTREM2-analyses were measured at the same laboratory using a sensitive TREM2 ELISA. The samples were analyzed at the same time but not concomitantly. In brief, the plates were coated with an anti-humanTREM2 polyclonal capture antibody (AF1828, R&D Systems, Minneapolis, MN, USA) and sTREM2 was detected by a mouse anti-human TREM2 monoclonal HRP-conjugated antibody (SEK11084, Sino Biologics, Beijing, China). Each sample was analyzed in duplicate and two CSF samples were included as internal standards in the assays to control for inter-day variability. For details of the protocol, see the previous publication^23^.

### Statistics

For statistical analyses, the IBM SPSS version 25 (IBM, Armonk, NY, USA) was employed. Demographic and clinical continuous variables were compared using one-way ANOVA, and for comparisons between categorical data, Pearson’s χ^2^ test was applied. If data did not fit test assumptions of normality distribution, nonparametric tests were used. The Mann–Whitney U test and Kruskal–Wallis test were used for group comparisons, and correlations were investigated with Spearman’s rank correlation coefficient. The level of sTREM2 was transformed using natural logarithm (LN) to limit the effect of outliers and to approximate normal distribution. Univariate linear regression analyses were carried out with sTREM2 as the dependent variable and age, gender, diagnoses, MMSE, and APOE genotype included as independent variables. As the CSF core AD biomarkers for the patients and the cognitively unimpaired were analyzed in two different laboratories, Aβ_42_, and P-tau were included after dichotomization and classified as positive or negative according to the laboratories’ references when comparing patients and cognitively unimpaired.

Age, gender, Aβ_42_, P-tau, and variables with a *p* value below 0.2 were included in multiple regression models. The level of significance was set at *p* ≤ 0.05. As the cognitively unimpaired were older than the patients, covariance analyses (ANCOVA) were performed to control for the influence of age on the values of sTREM2, both based on the clinical diagnoses and the AT classification. Graphical illustrations were created with GraphPad Prism8 (GraphPad Software, La Jolla, CA, USA).

### Ethics approval and consent to participate

All patients, their family caregivers, and cognitively unimpaired controls gave their written consent for participation. The study was approved by the regional Ethics Committee for medical research in the South-East of Norway (REK, 2011/2052 and REK 2017/371) and the Data Protector Officer at our institution.

### Consent for publication

Not applicable.

## Results

### Characteristics

The patients included in the study at the memory clinics were slightly younger than the cognitively unimpaired: 70.3 (SD 6.5), range 49–84, versus 72.3 (SD 6.0), range 64–89, *p* = 0.005. They also had fewer years of education: 12.0 (SD 3.7) versus 14.1 (SD 3.5), *p* < 0.001. No difference in gender distribution between the groups was found (*p* = 0.09). As expected more patients than cognitively unimpaired were *APOE ε4* positive (73.9% vs. 38.0%) and the patients performed significantly worse on cognitive tests. Of the 299 memory clinic patients, 62 (20.7%) were clinically diagnosed with MCI due to AD and 237 (79.3%) patients with dementia due to AD, whereas 11 (17.7%) AD-MCI and 59 (24.9%) AD dementia patients had AD mixed with cerebrovascular disease. The mean MMSE score in the AD-MCI group was 26.2 (SD 3.2), in the AD dementia group 22.5 (SD 4.5), and in the cognitively unimpaired group 29.2 (SD 0.9). Characteristics and differences between the AD-MCI patients, AD dementia patients, and the cognitively unimpaired are described in Table 1.

**Table 1:** Characteristics of the cohort.

	AD-MCI patients (N = 62)	AD dementia patients (N = 237)	Cognitively unimpaired (N = 113)	*p*
**Patient characteristics**				
Age [mean (SD)]	71.0 (5.4)	70.1 (6.8)	72.3 (6.0)	**0.01a**
Women [n (%)]	36 (58.1)	135 (57)	54 (47.8)	0.23b
Education (mean [SD])	13.0 (4.0)	11.7 (3.5)	14.1 (3.5)	**< 0.001a**
*APOE 4 *positive (n [%])**	39 (68.4)	156 (74.6)	41 (38.0)	**< 0.001b**
**Cognition**				
MMSE (mean [SD])	26.2 (3.2)	22.5 (4.5)	29.2 (0.9)	**< 0.001c**
CDT accepted (n [%])	61 (82.9)	105 (46.1)	108 (95.6)	**< 0.001b**
TMT A ≥ -2 SD (n [%])	51 (85.0)	127 (59.1)	106 (93.8)	**< 0.001b**
TMT B ≥ -2 SD (n [%])	41 (70.7)	73 (36.3)	105 (92.9)	**< 0.001b**
**CSF biomarkers**				
sTREM2 ng/ml (mean [SD])	9.9 (4.5)	9.5 (4.8)	8.8 (3.6)	0.31c
Amyloid β_42_ pg/ml (mean [SD])	628.6 (220.3)	556.6 (168.3)	705.2 (206.8)	*
Total tau pg/ml (mean [SD])	572.2 (264.3)	735.3 (381.6)	368.7 (149.2)	*
Phosphorylated tau pg/ml (mean [SD])	91.1 (38.3)	91.1 (38.3)	59.5 (20.1)	*

Bold values are statistically significant (*p* ≤ 0.05).

*MCI* mild cognitive impairment, *AD* Alzheimer's disease, *MMSE* mini mental state examination, *CDT* clock drawing test, *TMT* trail making test A and B.

a = one-way ANOVA, b = Chi square, c = Kruskal–Wallis, * = comparison not possible due to inter-laboratory variability, % = valid percent without missing, ** = missing genotype data in n = 38.

### CSF sTREM2 levels in relation to clinical presentation and biological profiles

**Figure 1:**
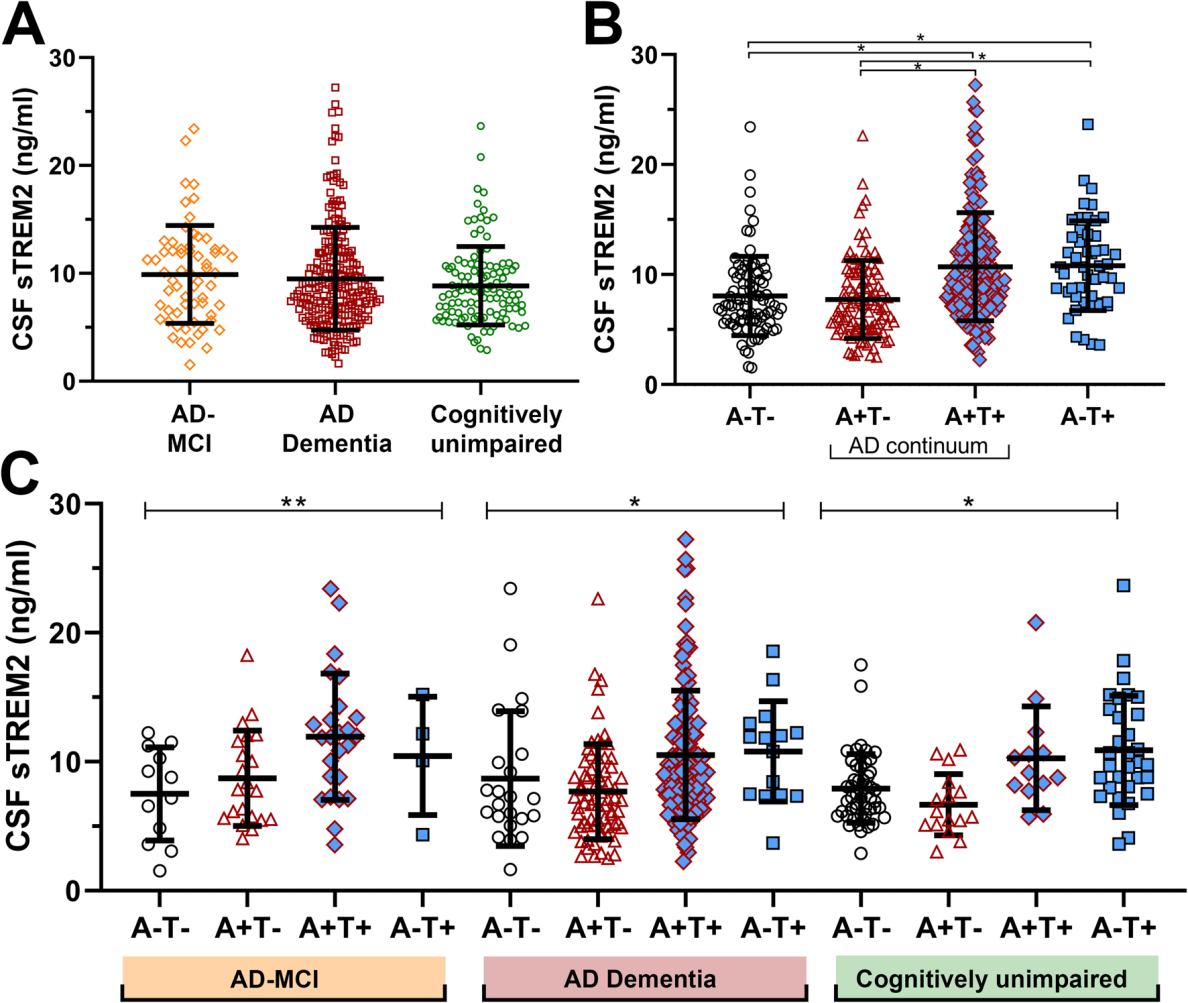
CSF sTREM2 within clinical presentation groups and AT categories. (**A**) Cerebrospinal fluid (CSF) sTREM2 levels did not relate to the AD clinical presentation. There was no difference in the CSF sTREM2 levels when comparing the cognitively unimpaired with patients having a clinical presentation of mild cognitive impairment (MCI) or dementia due to Alzheimer’s disease (AD) (*p* = 0.31, Kruskal–Wallis test, n = 113, n = 62, n = 237, respectively). (**B**) CSF sTREM2 related to AT status. When all individuals included were stratified according to the AT classification CSF sTREM2 was higher among those with biomarker data suggestive of tau pathology (T) *p < 0.001 obtained by Mann–Whitney-test. (**C**) The stratification according to the AT system within the clinical groups also showed higher CSF sTREM2 among those with biomarker data suggestive of tau pathology (AD-MCI **p = 0.02, AD dementia * and cognitively unimpaired *p < 0.001, *p* values obtained by Kruskal–Wallis test). Data are presented with individual values with smaller and larger lines representing the mean and standard deviation, respectively.

No differences were found in mean sTREM2 levels either between the patients (9.6 ng/ml [SD 4.7]) and the cognitively unimpaired (8.8 ng/ml [SD 3.6], *p* = 0.27, Mann–Whitney U test [MW]). There were also no differences in mean sTREM2 levels between the different AD clinical stages MCI and dementia (9.9 ng/ml [SD 4.5] and 9.5 ng/ml [SD 4.8], *p* = 0.31, MW) or between patients with probable AD (9.8 ng/ml [SD 4.9]) and AD mixed with cerebrovascular disease (8.9 ng/ml [SD 4.0], *p* = 0.31, respectively). See Fig. 1A. This remained after correction for age differences.

The AT(N) classification system were applied as described by Jack et al.^3^ with CSF measures of amyloid-β_1-42,_ phosphorylated tau and total tau used for classification of A, T and (N), respectively (See Supplementary Table 1). Due to few patients in some of the groups when adding (N), the main focus was restricted to the AT categorization.

In the further analyses, the patients and cognitively unimpaired were then stratified according to the AT classification^3^. When dividing the whole cohort into two groups according to positive or negative CSF measures of Aβ-deposition (A+ and A−), independent of clinical diagnoses, no difference was found in sTREM2 values (9.5 ng/ml [SD 4.6] vs. 9.1 ng/ml [SD 4.0], *p* = 0.60, MW). In contrast, dividing the cohort into two groups according to positive or negative CSF measures of tau-pathology (T+ and T−), a significant difference was found, with a higher mean value in the T+ group (10.7 ng/ml [SD 4.9] vs. 7.7 ng/ml [SD 3.6], *p* < 0.001, MW). This difference remained after adjusting for age. Further division into four groups accounting for overlap between the pathologies; one group with all negative biomarkers (A−T−) and three groups with one or two positive biomarkers, A+T−, A+T+ and A−T+, a significant difference was found with the lowest means in the T− groups, and the highest means in the T+ groups. Even after taking into account the clinical stages, the same pattern was found. For results, see Table 2 and Fig. 1 B and C.

**Table 2:** AT classification.

	Total N	A−T−	A+T−	A+T+	A−T+	*p*
The whole cohort (n [%])	412 (100)	87 (21.1)	109 (26.5)	165 (40.0)	51 (12.4)	
sTREM2 ng/ml (mean [SD])		8.1 (3.6)	7.7 (3.6)	10.7 (4.9)	10.8 (4.1)	**< 0.001a**
AD-MCI patients (n [%])	62 (100)	12 (19.4)	21 (33.9)	25 (40.3)	4 (6.5)	
sTREM2 ng/ml (mean [SD])		7.5 (3.6)	8.7 (3.7)	11.9 (4.9)	10.5 (4.6)	**0.02a**
AD dementia patients (n [%])	237 (100)	23 (9.7)	72 (30.4)	127 (53.6)	15 (6.3)	
sTREM2 ng/ml (mean [SD])		8.6 (5.2)	7.7 (3.7)	10.5 (5.0)	10.8 (3.9)	**< 0.001a**
Cognitively unimpaired (n [%])	113 (100)	52 (46.0)	16(14.2)	13 (11.5)	32 (28.2)	
sTREM2 ng/ml (mean [SD])		7.9 (2.7)	6.7 (2.4)	10.3 (4.0)	10.9 (4.2)	**< 0.001a**

Bold values are statistically significant (*p* ≤ 0.05).

*MCI* mild cognitive impairment, *AD* Alzheimer's disease.

a = Kruskal–Wallis test, % = valid percent without missing.

At last, comparing the A−T− cognitively unimpaired group with the A+T+AD-MCI and AD dementia patients there was a significant difference in the mean sTREM2 levels was, (7.9 ng/ml [SD 2.7] vs. 10.8 [SD 5.0], respectively, *p* < 0.001, MW).

### Regression analyses

In the regression analyses, the patients and the cognitively unimpaired were analyzed separately. Univariate analyses of patients with clinical AD (both MCI and dementia) showed a significant association between sTREM2 and age (β = 0.30, *p* < 0.001) and between sTREM2 and P-tau (β = 0.38, *p* < 0.001). No association was found between sTREM2 and clinical diagnoses, Aβ_42,_ gender or *APOE ε4* genotype. In the multiple regression analyses, the association with age (β = 0.29, *p* < 0.001) and P-tau (β = 0.39, *p* < 0.001) remained significant and explained 23% of the variance (see Table 3). The same analyses of the cognitively unimpaired also resulted in the same positive associations (see Table 4). Separate analyses of all the A−T− individuals confirmed the association between sTREM2 and age (results not shown). Finally, an analysis of A−T− cognitively unimpaired and A+T+AD-MCI and AD dementia patients resulted in the same association being found, explaining 11% of the variance (see Table 5).

**Table 3:** Multiple linear regression – LN sTREM2, the patient cohort (n = 299).

	Univariate			Multivariate	
	Standardized β	*p*	R square, adjusted	Standardized β	*p*
Diagnoses (MCI = 1, AD dementia = 2)	− 0.04	0.54	− 0.002		
Age	0.30	**< 0.001**	0.09	0.29	**< 0.001**
Gender (0 = women, 1 = men)	0.07	0.25	0.001	0.08	0.14
Education	− 0.11	0.56	0.009		
MMSE	− 0.04	0.53	− 0.002		
*APOE ε4* genotype (neg = 0, pos = 1)	− 0.02	0.81	− 0.004		
Amyloid β42	0.05	0.40	− 0.01	0.02	0.70
Phosphorylated tau	0.38	**< 0.001**	0.15	0.39	**< 0.001**
R square, adjusted					0.23

Bold values are statistically significant (*p* ≤ 0.05).

*MCI* mild cognitive impairment, *AD* Alzheimer's disease, *MMSE* mini mental state examination.

**Table 4:** Multiple linear regression—LN sTREM2, cognitively unimpaired controls (n = 113).

	Univariate			Multivariate	
	Standardized β	*p*	R square	Standardized β	*p*
Age	0.27	**0.004**	0.07	0.18	**0.03**
Gender (0 = women, 1 = men)	0.05	0.62	− 0.007	0.07	0.37
Education	− 0.10	0.23	0.004		
*APOE ε4* genotype (neg = 0, pos = 1)	0.10	0.29	0.001		
Amyloid β42	− 0.11	0.23	0.004	0.12	0.14
Phosphorylated tau	0.41	**< 0.001**	0.16	0.35	**< 0.001**
R square, adjusted					0.20

Bold values are statistically significant (*p* ≤ 0.05).

**Table 5:** Multiple linear regression—LN sTREM2, A−T− cognitively unimpaired (n = 52) versus A+T+ AD MCI and dementia patients (n = 152).

	Univariate			Multivariate	
	standardized β	P	R square	Standardized β	P
AT-classification (A−T− = 1, A+T+ = 2)	0.25	**< 0.001**	0.06	0.39	**< 0.001**
Age	0.24	**0.001**	0.05	0.16	**0.03**
Gender (0 = women, 1 = men)	0.03	0.65	− 0.004	0.07	0.34
Education	− 0.14	**0.05**	0.01	− 0.09	0.25
MMSE	− 0.11	0.14	0.006	0.12	0.21
*APOE ε4* genotype (neg = 0, pos = 1)	0.13	0.08	0.01	− 0.01	0.88
R square, adjusted					0.11

Bold values are statistically significant (*p* ≤ 0.05).

*MMSE* mini mental state examination.

## Discussion

In this study of memory clinic patients with AD and cognitively unimpaired controls, the sTREM2 level did not differ between the patients and the cognitively unimpaired or between clinical presentation (AD-MCI and AD dementia). Moreover, regression analyses found no associations with CSF Aβ_42_, *APOE ε4 *genotype or gender. The only factors independently associated with sTREM2 were increasing age and tauopathy as reflected by higher sTREM2 levels in the T+ groups overall and within the clinical groups including the cognitively unimpaired.

While most studies find a tendency for a higher sTREM2 level in AD dementia ^21–24,31^, the results at the MCI stage are less consistent. In one study, MCI patientswith a positive AD biomarker profile were found to present with the highest sTREM2 levels and notably MCI patients without such a profile to present with a lower level indicating the importance of biomarker-assisted diagnosis^20^. In contrast, another biomarker-selected study found significantly higher sTREM2 levels in AD dementia than in MCI, but both groups presented with higher levels relative to the control group^31^. Yet another study found a higher sTREM2 level in both AD dementia and MCI compared to healthy controls^24^. A meta-analysis from 2018, including cross-sectional cohorts and case–control studies, found significantly increased sTREM2 levels in all stages of AD compared to the controls, with the highest levels in MCI^39^. The majority of studies indicate that sTREM2 is an inflammatory marker of the early stages of AD^23,39,40^. However, few studies have stratified both clinical patient groups and their biomarker profile. Indeed, when including only AD biomarker-negative cognitively unimpaired and AD biomarker-positive patients we also found higher sTREM2 levels in the patient group. Further, including patients with an AD clinical diagnosis and cognitively unimpaired irrespective of the AD biomarker profile allowed us to compare AT effects within the clinical groups confirming the assumption that sTREM2 relates strongly to tauopathy irrespective of the clinical presentation.

In the present study, the association between sTREM2 and tauopathy (T+) was independent of amyloid pathology which is consistent with other studies^21,22,25,32^. Our data also reproduce Suárez-Calvet et al.’s finding from the Alzheimer’s Disease Neuroimaging Initiative (ADNI) cohort that the lowest levels were in participants who were only A+(A+T−(N−)), compared to all other biomarker profiles^25^. Likewise, those with tangle pathology (A−T+ and A+T+) presented with the highest sTREM2 levels. Additionally, we analyzed sTREM2 relationships with the different clinical presentations at the MCI or dementia stage, probable AD/mixed cerebrovascular disease (based on the NIA/AA criteria), or with cognitively unimpaired controls. This allowed us to determine that there were no differences in sTREM2 levels between these clinical groups.

We speculate increased CSF sTREM2 being due to microglial activation in response to tauopathy in general and to what is presumably A+ facilitated tau pathology in AD^41^. Although TREM2 may bind Aβ and exert effects e.g. stimulating Aβ-clearance as shown in mouse brain^42,43^ CSF sTREM2 may not reflect such activation in the absence of downstream induced tauopathy. TREM2 serves regulatory checkpoint functions to various external stimuli^44^. Interestingly in the absence of TREM2, with elimination of this checkpoint function, neuritic dystrophy and phospho-tau immunoreactivity increased around senile plaques in transgenic mice seeded with human tau-aggregates^45^. Although studies with genetically manipulated models like here a knock-out model^45^ and sporadic AD patients can create vastly different outcomes^46,47^ the findings both link the same pathogenic processes; tauopathy with microgliosis/TREM2. Although the results seem opposite, they may not be as biological processes create responses by functioning in networks.

In a recent clinical study including many of the same cognitively unimpaired controls as in the current study, Halaas et al. found an association between sTREM2 and neurodegeneration measured by MRI ^48^. This significant association between sTREM2 and brain atrophy (especially lateral temporal lobe atrophy and hippocampal atrophy) was highest among those with increased levels of P-tau compared with those with brain atrophy without P-tau pathology^48^. Due to the high correlation between P-tau (T) and T-tau (N) we could not include the results of T-tau in the present study. With an advanced age, there is an increased prevalence of suspected non-Alzheimer disease pathophysiology (SNAP; N+), a group that includes multiple different pathologies such as cerebrovascular disease, α-synucleinopathies, argyrophilic grain disease, TDP-43 proteinopathies and hippocampal sclerosis^49^ and the related primary age-related tauopathy (PART; A−/T+)^50^. Both SNAP and PART relate to pathological changes characterized by neurofibrillary tangles (PART) and neurodegeneration (SNAP)^49,50^. In both conditions, Aβ-deposition is minimal, the cognitive impairment often modest and the clinical presentation similar to that of AD. In our study, 16.0% of the AD dementia patients and 25.8% of the AD-MCI patients were A−, whereas 12.8% of the AD-MCI and AD dementia patients were A−T+ with non-AD pathological changes that may be due to PART. In the case of the cognitively unimpaired controls, the only exclusion criteria were abnormal results on the cognitive tests. When classifying them according to the AT criteria, only 46.0% had normal AD biomarkers (A−T−), and non-AD pathological changes (A−T+) were found among 28.2% of the cognitively unimpaired presumably related to brain reserve capacity. As speculated, these findings may explain why differences in sTREM2 between the clinical groups were only found between A−T− cognitively unimpaired and A+T+ patients.

The latter observation is consistent with data in the longitudinal study of Halaas et al. with no association between baseline CSF sTREM2 and cognitive decline among cognitively unimpaired individuals followed for 4 years^48^. Transiently increased CSF sTREM2 by secondary tauopathy could reflect a protective microglial response at early stage AD. In another recently published longitudinal study, an association between an increased sTREM2/P-tau ratio and decreased memory decline was found^33^. The suitability of sTREM2 as a prognostic marker would thus be interesting to further explore.

Aging is the leading risk factor for dementia, including AD dementia. In accordance with most studies, we found a positive association between sTREM2 and age^20,23,25^. However, some find this association only in the control groups^23^. Nordengen et al. did not find any association between age and CSF sTREM2 among unimpaired controls (n = 36) when controlling for T-tau. However, their control group had a mean age of 61.1 (SD 9.2) years and were all A−T−(N−)^31^. In our study, the association with age remained in A−T− cognitively unimpaired controls (Table 5). From animal studies, it is known that gliosis increases with aging. Thus CSF sTREM2, which is a quite specific microglial marker, may reflect age-dependent increased microglial senescence^51^.

The findings in our study support the hypothesis of sTREM2 being a microglial marker related to tauopathy and brain aging. Its value as a biomarker along the Alzheimer’s disease continuum is mainly linked to tauopathy, which by unclear mechanisms presumably is secondary to Aβ-aggregation in many cases of Alzheimer’s disease^41^. The TREM2-related microglial activation seems to be part of the pathophysiological mechanisms activated in brain aging and brain atrophy rather than being an inflammatory marker connected to the presumed early pathogenic processes involving Aβ along the Alzheimer’s continuum^48^. It might be that sTREM2 reflects microglial activity associated with neurite changes around amyloid deposits, since its formation also depends on proteolytic activity. CSF sTREM2 seems less suitable for classification in dementia diagnostics^10,24^, and like many other biomarkers becomes less valuable with increasing patient age due the increased prevalence of several independent pathogenic processes in the brain.

### Strengths

The cohort in the study reflects biomarker unselected patients referred to a memory clinic for a diagnostic workup. Major strengths in this study are the relatively large patient cohort with an age range from 49 to 84 years of age, and the standardized comprehensive assessment, including neurocognitive examination, physical examinations (e.g., blood and CSF samples), and brain imaging. Another strength is the group of relatively old cognitively unimpaired controls (age range 64–89 years) who was comprehensively assessed. People with decreased performance on the cognitive tests after two years were excluded.

### Limitations

A limitation is that it was not possible to include the results on brain imaging when classifying the cohort according to the AT(N) classification. It was based on the CSF core biomarkers only as suggested by Jack et al.^3^. Due to a high correlation between P-tau and T-tau, including (N) in the regression analyses provided no further insight. Another limitation was that the core biomarkers were analyzed in two different laboratories, which prevented certain statistical comparison. Finally, the cross-sectional design did not allow examination of cognitive changes with time in relation to biomarker levels of individual patients.

## Conclusion

In this study, an association between increased levels of sTREM2, age and tauopathy was found regardless of clinical presentation. sTREM2 was found not suitable for differentiating between the cognitively unimpaired and those afflicted by disease in this population. Our results support the hypothesis of sTREM2 mainly being a microglial activation marker connected to tauopathy and brain aging, irrespective of clinical presentation, and an increase of it along an AD continuum being indicative of secondary tauopathy and pronounced neurite dystrophy around Aβ-deposits in the brain.

## Supplementary information

Supplementary file1

## Data Availability

Legal restrictions, imposed by the registry owners and the ethical committee, prevent us from publicly sharing our identified data set due to sensitive patient information. Data may be requested from the Norwegian Registry of Persons Assessed for Cognitive Symptoms (contact: post@aldringoghelse.no). As for the cognitively unimpaired controls, data are available upon reasonable request to the authors. For both cohorts, availability is dependent on approval from the Regional Ethics Committee for medical research in the South-East of Norway (contact: post@helseforsikring.etikkom.no).

## Abbreviations

A: Amyloid pathology
Aβ_42_: Amyloid β_1-42_
AD: Alzheimer’s disease
CSF: Cerebrospinal fluid
MCI: Mild cognitive impairment
MMSE: Mini mental status examination
N: Neurodegeneration
PART: Primary age-related tauopathy
P-tau: Phosphorylated tau
SNAP: Suspected non-Alzheimer disease pathophysiology
sTREM2: Soluble triggering receptor expressed on myeloid cells 2
T: Phosphorylated tau pathology
T-tau: Total tau
