# Supplementary material for: Cerebrospinal fluid sTREM2 in Alzheimer’s disease: comparisons between clinical presentation and AT classification
Source: Sci Rep. 2020 Sep 28;10:15886. doi: 10.1038/s41598-020-72878-8 (PMC7522273; doi:10.1038/s41598-020-72878-8)
Supplement: Supplementary file 1 — Supplementary file1 [file 41598_2020_72878_MOESM1_ESM.pdf]

Supplementary

Cerebrospinal fluid sTREM2 in Alzheimer's disease - comparisons between clinical presentation and AT classification

Anne-Brita Knapskog* [1], Kristi Henjum[2,3], Ane-Victoria Idland[1,4], Rannveig Sakshaug Eldholm[5,6], Karin Persson[1,7], Ingvild Saltvedt[5,6], Leiv Otto Watne[1], Knut Engedal[1,7], Lars N.G. Nilsson[2].

[1] Department of Geriatric Medicine, Oslo University Hospital, Oslo, Norway

[2] Department of Pharmacology, Institute of Clinical Medicine, University of Oslo and Oslo University Hospital, Oslo, Norway

[3] Department of Geriatric Medicine, Institute of Clinical Medicine, University of Oslo, Oslo, Norway

[4] Institute of Basic Medical Sciences, University of Oslo, Oslo, Norway

[5] Department of Neuromedicine and Movement Science, Norwegian University of Science and Technology (NTNU), Trondheim,

[6] Department of Geriatrics, St Olavs Hospital, University Hospital of Trondheim,

[7] Norwegian National Advisory Unit on Ageing and Health, Vestfold Hospital Trust,

Supplementary Table 1. AT(N) classification

	A-T-(N-)	A+T-(N-)	A+T+(N-)	A+T+(N+)	A+T-(N+)	A-T+(N-)	A-T-(N+)	A-T+(N+)
The whole cohort (n [%])	75 (18.2)	58 (14.1)	7 (1.7)	158 (38.3)	51 (12.4)	2 (0.5)	12 (2.9)	49 (11.9)
sTREM2 ng/ml (mean [SD])	7.9 (3.6)	6.8 (2.9)	7.5 (1.2)	10.9 (5.0)	8.8 (4.0)	12.2 (0.1)	9.2 (3.4)	10.8 (4.2)
AD-MCI (n [%])	10 (16.1)	12 (19.4)	0	25 (40.3)	9 (14.5)	1 (1.6)	2 (3.2)	3 (4.8)
sTREM2 ng/ml (mean [SD])	7.0 (3.7)	8.1 (4.2)		11.9 (4.9)	9.5 (3.0)	12.2	10.3 (1.4)	9.9 (5.4)
AD dementia patients (n [%])	19 (8.0)	32 (13.5)	5 (2.1)	122 (51.5)	40 (16.9)	1 (0.4)	4 (1.7)	14 (5.9)
sTREM2 ng/ml (mean [SD])	8.9 (5.5)	6.4 (2.5)	7.5 (0.8)	10.6 (5.0)	8.7 (4.2)	12.2	7.9 (4.1)	10.7 (4.0)
Cognitively unimpaired (n [%])	46 (40.7)	14 (12.4)	2 (1.8)	11 (9.7)	2 (1.8)	0	6 (5.3)	32 (28.3)
sTREM2 ng/ml (mean [SD])	7.7 (2.5)	6.6 (2.2)	7.2 (2.4)	10.8 (4.1)	7.2 (4.8)		9.7 (3.5)	10.9 (4.2)

Comparison not possible due to few or no participants in some groups

Supplementary Table 2. A+T+N+ versus A+T-N+

	**A+T+N+**	**A+T-N+**	**P**
The whole cohort (n [%])	158 (38.3)	51 (12.4)	
sTREM2 ng/ml (mean [SD])	10.9 (5.0)	8.8 (4.0)	0.007a
AD-MCI (n [%])	25 (40.3)	9 (14.5)	
sTREM2 ng/ml (mean [SD])	11.9 (4.9)	9.5 (3.0)	0.18a
AD dementia patients (n [%])	122 (51.5)	40 (16.9)	
sTREM2 ng/ml (mean [SD])	10.6 (5.0)	8.7 (4.2)	0.02a
Cognitively unimpaired (n [%])	11 (9.7)	2 (1.8)	
sTREM2 ng/ml (mean [SD])	10.8 (4.1)	7.2 (4.8)	*

a = Mann-Whitney test, *comparison not possible due to few patients in one group
